# Accuracy of lung and diaphragm ultrasound in predicting infant weaning outcomes: a systematic review and meta-analysis

**DOI:** 10.3389/fped.2023.1211306

**Published:** 2023-09-06

**Authors:** Yang Gao, Hong Yin, Mei-Huan Wang, Yue-Hua Gao

**Affiliations:** ^1^ Department of Ultrasound, Shandong Provincial Maternal and Child Health Care Hospital, Jinan, China; ^2^ Department of Ultrasound, Shandong Provincial Hospital Affiliated to Shandong First Medical University, Jinan, China

**Keywords:** lung ultrasound, diaphragm ultrasound, weaning, mechanical ventilation, pediatric critical care, endotracheal extubation

## Abstract

**Background:**

Although lung and diaphragm ultrasound are valuable tools for predicting weaning results in adults with MV, their relevance in children is debatable. The goal of this meta-analysis was to determine the predictive value of lung and diaphragm ultrasound in newborn weaning outcomes.

**Methods:**

For eligible studies, the databases MEDLINE, Web of Science, Cochrane Library, PubMed, and Embase were thoroughly searched. The Quality Assessment of Diagnostic Accuracy Studies (QUADAS−2) method was used to evaluate the study's quality. Results were gathered for sensitivity, specificity, diagnostic odds ratio (DOR), and the area under the curve of summary receiver operating characteristic curves (AUSROC). To investigate the causes of heterogeneity, subgroup analyses and meta-regression were conducted.

**Results:**

A total of 11 studies were suitable for inclusion in the meta-analysis, which included 828 patients. The pooled sensitivity and specificity of lung ultrasound (LUS) were 0.88 (95%CI, 0.85–0.90) and 0.81 (95%CI, 0.75–0.87), respectively. The DOR for diaphragmatic excursion (DE) is 13.17 (95%CI, 5.65–30.71). The AUSROC for diaphragm thickening fraction (DTF) is 0.86 (95%CI, 0.82–0.89). The most sensitive and specific method is LUS. The DE and DTF were the key areas where study heterogeneity was evident.

**Conclusions:**

Lung ultrasonography is an extremely accurate method for predicting weaning results in MV infants. DTF outperforms DE in terms of diaphragm ultrasound predictive power.

## 1. Introduction

Mechanical ventilation (MV) has been widely employed to enhance outcomes in pediatric intensive care unit (PICU) and respiratory care in very preterm newborns (1, 2). However, weaning has long been a cause of concern. Prolonged MV is related with infant ventilator-associated lung injury and neurodevelopmental impairment, whereas premature weaning is associated with extubation failure (EF) and an increase risk of reintubation (3, 4). Moreover, many studies have linked EF and subsequent reintubation to increased mortality and bronchopulmonary dysplasia (BPD) in premature newborns (5–7). Therefore, choosing the best weaning interval has a substantial clinical impact on the prognosis of newborns with MV.

There are currently no standard guidelines to guide clinical weaning of infants with MV (8), and weaning timing is primarily determined by the attending physician based on clinical evaluation, ventilator parameters, and blood gas results (9), resulting in approximately 30% of premature infants failing extubation (10). As a result, new precise indications for predicting successful weaning timing in children with MV are required.

Because of its non-invasive, convenient, and real-time features, ultrasound is appropriate for bedside assessment of MV patients in the ICU (11, 12). Diaphragmatic function and pulmonary ventilation status are two components of ultrasound assessment of whether a patient can be weaned, with the former proven by the diaphragmatic excursion (DE) and diaphragm thickening fraction (DTF) (13), and the latter by the lung ultrasound score (LUS) (14). These three signs have been demonstrated to be accurate predictors of weaning in adult MV patients (15). Short DE distances and high DTF scores indicate poor diaphragm function, whereas high LUS suggests inadequate pulmonary ventilation, implying a significant risk of EF (16). However, because infant physiology and anatomy differ from those of adults, more research is needed to determine whether ultrasound technology can be used to anticipate weaning in infants with MV.

The purpose of this meta-analysis is to establish the accuracy of three diaphragm and lung ultrasound indicators: DE, DTF, and LUS in predicting weaning outcomes in infants with MV.

## 2. Methods

### 2.1. Literature search

Two researchers separately and methodically searched data from relevant papers published in MEDLINE, Web of Science, Cochrane Library, PubMed, and Embase from the time the databases were established until April 1, 2,023. Reference lists of relevant literature were also combed for any further relevant literature. The following MESH words were used in our search: [(“lung ultrasound” OR “diaphragm ultrasound” OR “LUS” OR “DE” OR “DTF”) AND (“MV” OR “weaning” OR “extubation” OR “discontinuation of mechanical ventilation” OR “disconnect of mechanical ventilation”) AND (“infants” OR “neonates” OR “children” OR “newborns”)].

### 2.2. Inclusion and exclusion criteria

The following are the inclusion criteria: (1) Infants receiving MV therapy who are getting ready to wean. (2) Patients who had diaphragm and/or lung ultrasounds prior to weaning. (3) Studies directly or indirectly offer true positive (TP), true negative (TN), false positive (FP), and false negative (FN) data to build 2 × 2 tables. The exclusion criteria are as follows: (1) Studies that are repeated or overlapped. (2) The data in the article is inadequate to create a 2 × 2 table. (3) research that is not original. Disagreements between the two researchers were resolved with the assistance of a third researcher.

### 2.3. Data extraction and quality assessment

Two researchers separately gathered TP, TN, FP, and FN from source literature data to produce a 2 × 2 form, and any inconsistencies were handled through deliberation. We only built data tables for weaning success due to the dichotomous nature of weaning events. Furthermore, various methodological data linked to research baseline features such as patient age, patient number, measurements and cutoff value, weaning success definition, and weaning failure definition were comprehensively retrieved. The QUADAS-2 tool was used to evaluate the four aspects of patient selection, flow and timing, index testing, and reference standards in all included studies (17). Utilizing Review Manager 5.3, the QUADAS-2 quality evaluation's bias and applicability concerns were rated as having low, high, or uncertain risk.

### 2.4. Statistical analysis

Pooled TP, TN, FP, and FN were used to compute sensitivity, specificity, positive likelihood ratio (PLR), negative likelihood ratio (*N*LR), diagnostic odds ratio (DOR), and corresponding 95% confidence intervals (95% CI) for diaphragm and lung ultrasound in MV infants. Furthermore, the summarized receiver operator characteristic (SROC) curve and area under the SROC curve (AUC) are utilized to assess the overall performance of ultrasound parameters (18).

We used *I*^2^ statistics and Q test for heterogeneity, and meta-regression to investigate sources of heterogeneity in sensitivity and specificity (19). Zones (6 zones were the most commonly reported zoning scheme); chance of offline success (70% average); cut-off (LUS ≥ 15); and whether or not the operation is blinded were research elements that investigated the accuracy of LUS. Additionally, the Deeks funnel plots were employed to calculate potential publication bias (20). This entire statistical procedure was carried out in Stata 16.0.

## 3. Results

### 3.1. Study selection and characteristics

**Figure 1:**
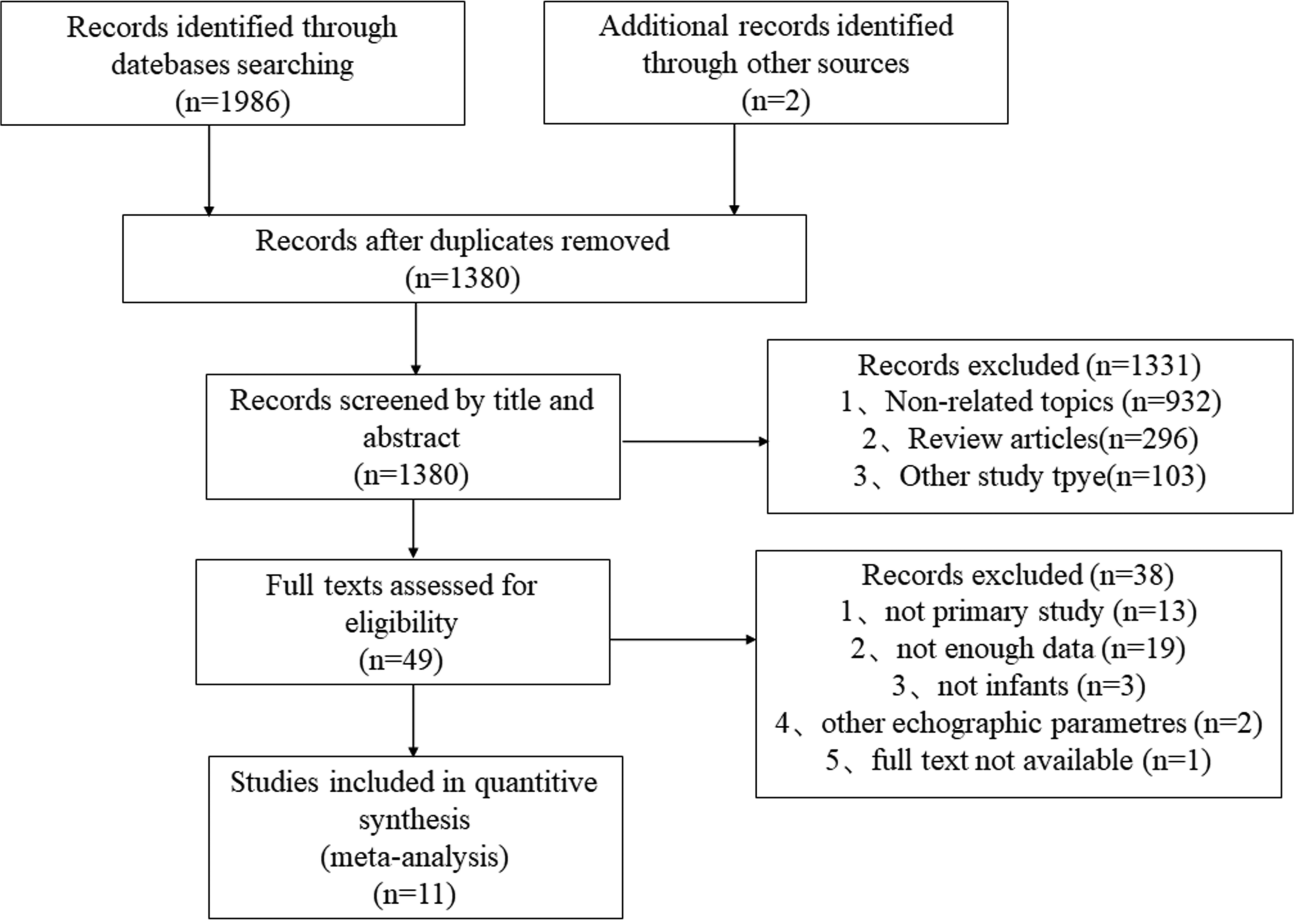
Flow diagram of the study selection process.

A detailed flow chart of our retrieved literature is shown in Figure 1. After deleting duplicate articles, we had 1,380 records after searching the original database for 1,986 articles. Following a review of the title and abstract, 1,331 records were excluded, and 38 records that did not fulfill the criteria were excluded after full text review. Finally, this meta-analysis contained 11 studies that met the inclusion and exclusion criteria (21c31).

Our study comprised 828 patients from 11 articles, with comprehensive baseline characteristics shown in Table 1. Four of the 11 studies were carried out in Egypt, four in China, two in Canada, and one in Turkey. Except for two unspecified trials (30, 31), the remaining nine were conducted in medical ICU. In addition, seven studies assessed the LUS indicator, four assessed the DE indicator, and four assessed the DTF indicator. Amrousy's study had two LUS cut-off points (24), but Jiang's study had four LUS cut-off points (31). One study looked at left and right DE (25), while the other looked at left and right DTF (29).

**Table 1:** Characteristics of included studies.

Study	Country	Setting	N	Age	Inclusion	Measurements and cutoff value	Weaning success definition	Weaning failure definition
Mohsen, 2022 (21)	Canada	NICU	45	SG: 25.5 ± 1.1;	Neonates with GA < 28 w, MV ≥ 12 h and ready for extubation	LUS score < 15	Extubation > 72 h without reintubation	Reintubation within 72 h
				FG:24.3 ± 0.6				
				(GA)				
Soliman, 2020 (22)	Egypt	NICU	66	30 (29–32)	Neonates supported with MV due to ARDS	LUS score < 11.5	NR	Reintubation
				(GA)				
Abdelmawla, 2022 (23)	Canada	NICU	39	SG: 31 ± 2.4;	Neonates supported with nCPAP	LUS score < 8	nCPAP was stopped and not restarted until discharge	NR
				FG: 29 ± 2.1				
				(GA)				
Amrousy, 2020 (24)	Egypt	NICU	80	SG: 13.2 ± 3.2; FG: 16.2 ± 4.5 (d)	Neonates with MV who were eligible for weaning	LUS score ≤ 4 & LUS score ≤ 6	NR	Reintubation or the need for invasive ventilatory support within 48 h after extubation
Bahga, 2021 (25)	Egypt	NICU	43	SG: 29.5 ± 1.7;	Preterm infants with MV and planned for weaning	Right DE ≥2.75 mm & Left DE ≥2.45 mm	Extubation >72 h without reintubation	NR
				FG: 29.2 ± 1.7				
				(GA)				
Xue, 2019 (26)	China	PICU	50	SG: 36.00 (15.00–84.00);	Children with MV >48 h and ready for weaning	DTF ≥ 21%	Maintain spontaneous breathing for >48 h	SBT failure
				FG: 42.00 (10.00–158.00)				
				(m)				
Abdel Rahman, 2020 (27)	Egypt	PICU	106	1 m to 170 m	Children with MV and planned for weaning	DTF ≥ 23.175% & DE ≥ 6.2 mm & LUS < 12	Extubation > 48 h without reintubation	Reintubation within 24–72 h
Gazi, 2022 (28)	Turkey	PICU	40	SG: 48.5 (87);	Children with MV >48 h	DTF ≥ 40.5% & DE ≥ 12.15 mm	Extubation > 48 h without reintubation	Reintubation or NIMV within 48 h
				FG: 20.5 (50)				
				(m)				
Yao, 2022 (29)	China	PICU	72	SG: 22.8 ± 6.8;	Children with MV >48 h	Right DTF ≥ 26.1% & Light DTF ≥ 20.7% & DE ≥ 8.08 mm	Extubation > 48 h without reintubation	SBT failure, reintubation or resu resumption of ventilatory support within 48 h
				FG: 23.7 ± 6.2				
				(m)				
Liang, 2021 (30)	China	NR	220	32.93 ± 3.50 (GA)	Infants with RDS, and MV support for ≥24 h	LUS score < 18	Extubation success	Reintubation within 48 h
Jiang, 2022 (31)	China	NR	67	SG: 31 (27, 36);	Infants with NRDS	LUS score < 8.5 & LUS score < 27.5 & LUS score < 25 & LUS score < 41	NR	MV with endotracheal intubation within 48 h
				FG: 29 (27, 33)				
				(GA)				

NICU, neonatal ICU; PICU, Pediatric ICU; NR, Not reported; SG, Successful weaning group; FG, Failed weaning group; GA, Gestational age, weeks; m, months; d, days; MV, Mechanical ventilation; ARDS, acute respiratory distresssyndrome; nCPAP, noninvasive continuous positive airway pressure; NRDS, neonatal respiratory distress syndrome; DE, Diaphragm excursion; DTF, Diaphragm thickening fraction; LUS, lung ultrasound; SBT, Spontaneous Breathing Trial.

Different studies identified different weaning criteria. Weaning success is defined primarily as sustaining spontaneous breathing >48 h without reintubation, whereas weaning failure is defined primarily as failing the Spontaneous Breathing Trial or requiring reintubation within 48 h.

### 3.2. Assessment of method quality and publication bias

**Figure 2:**
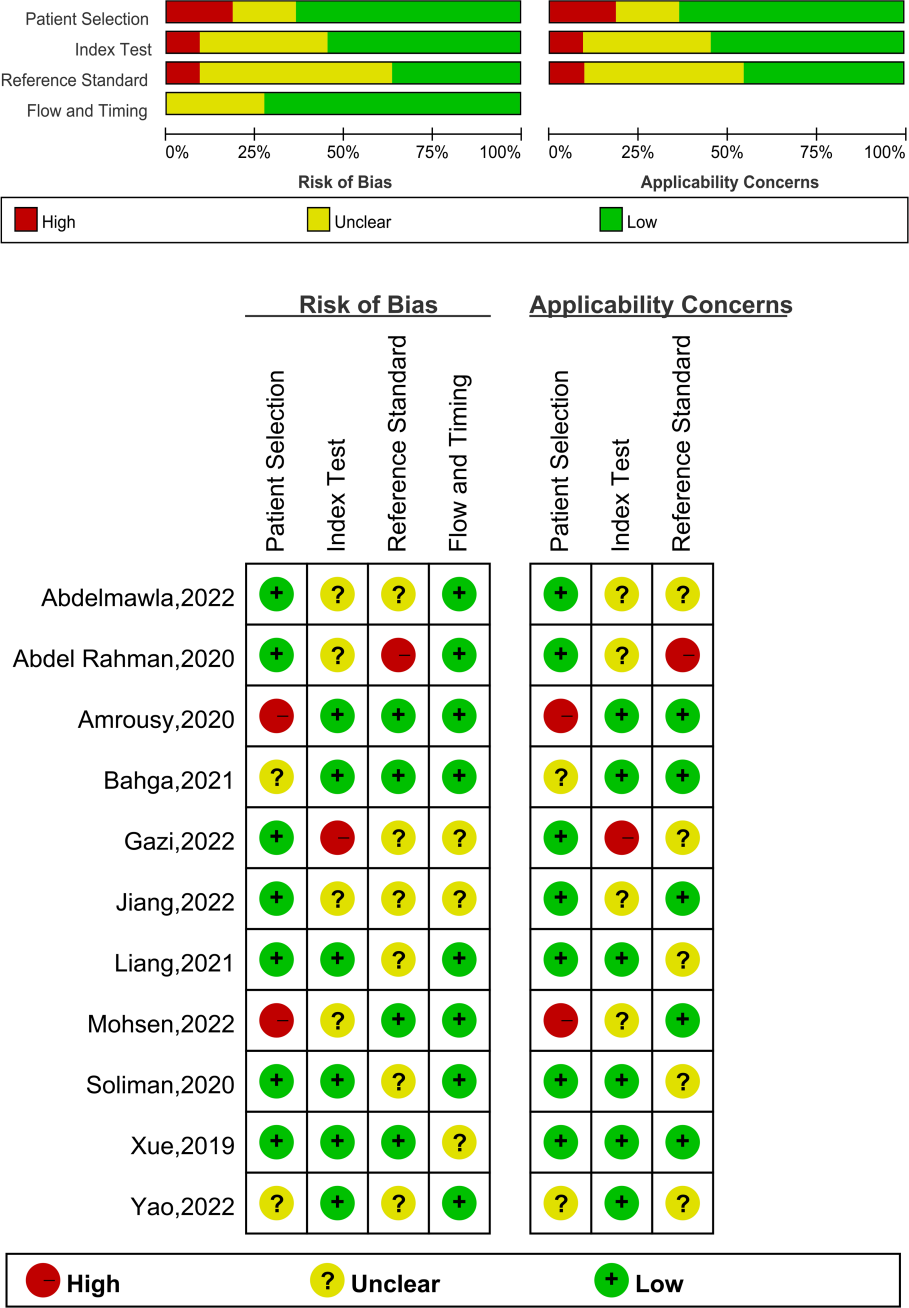
The graphical display of the evaluation of the risk of bias and concerns regarding the applicability of the selected studies.

The results of the QUADAS-2 tool assessing risk of bias and applicability concerns of the included studies were shown in Figure 2. The possibility of patient selection bias was mostly related to the fact that four studies included patients who were either eligible for weaning or planned to be weaned rather than patients who were randomly selected (21, 24, 25, 29). Index test poses a risk since they do not clearly reveal whether the ultrasound was performed without knowledge of the clinical outcomes (21, 23, 27, 28, 31). Furthermore, the reference standard poses a danger due to the low success rate of clinical weaning events and the omission to identify whether clinical weaning decisions were taken without knowledge of ultrasound findings (22, 27, 28, 29, 30, 31). The Deeks' test revealed no substantial publication bias in LUS, DE, or DTF, with all *P* values more than 0.05 (Supplementary Figure S1).

### 3.3. Diagnostic accuracy and heterogeneity

**Figure 3:**
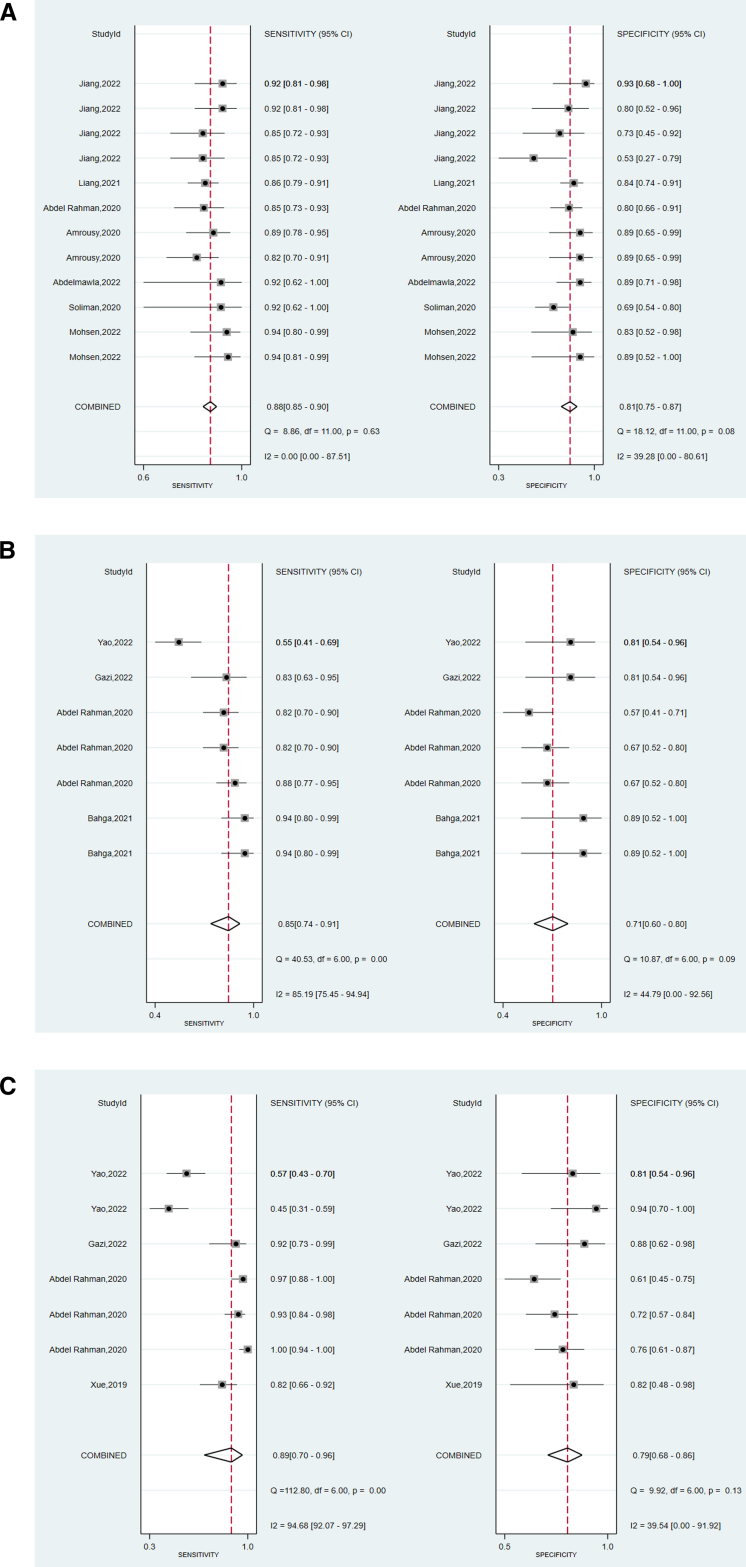
Forest plot of the pooled sensitivity and specificity of (**A**) LUS, (**B**) DE, and (**C**) DTF.

**Figure 4:**
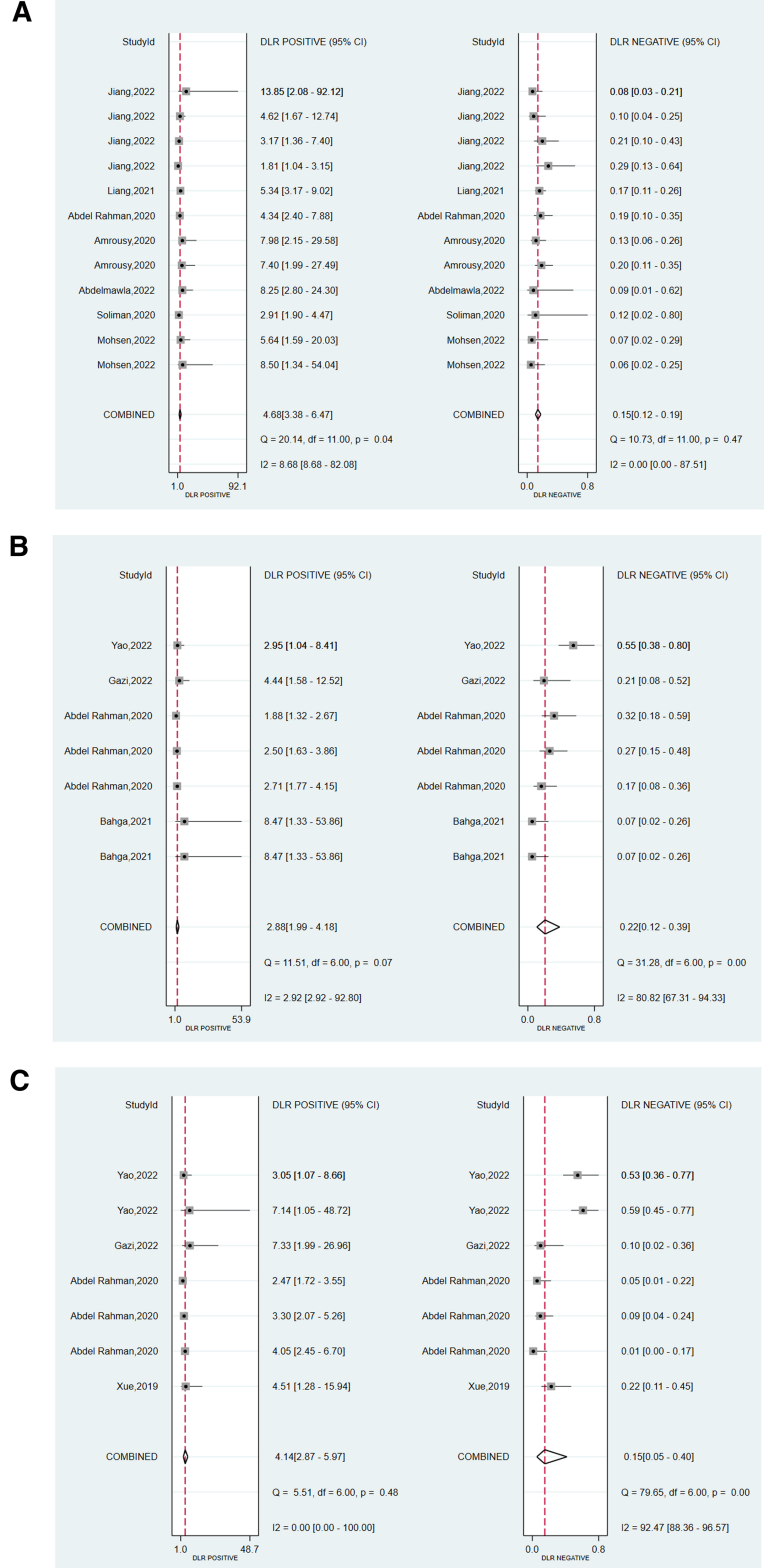
Forest plot of the pooled positive likelihood ratio (PLR) and negative likelihood ratio (*N*LR) of (**A**) LUS, (**B**) DE, and (**C**) DTF.

**Figure 5:**
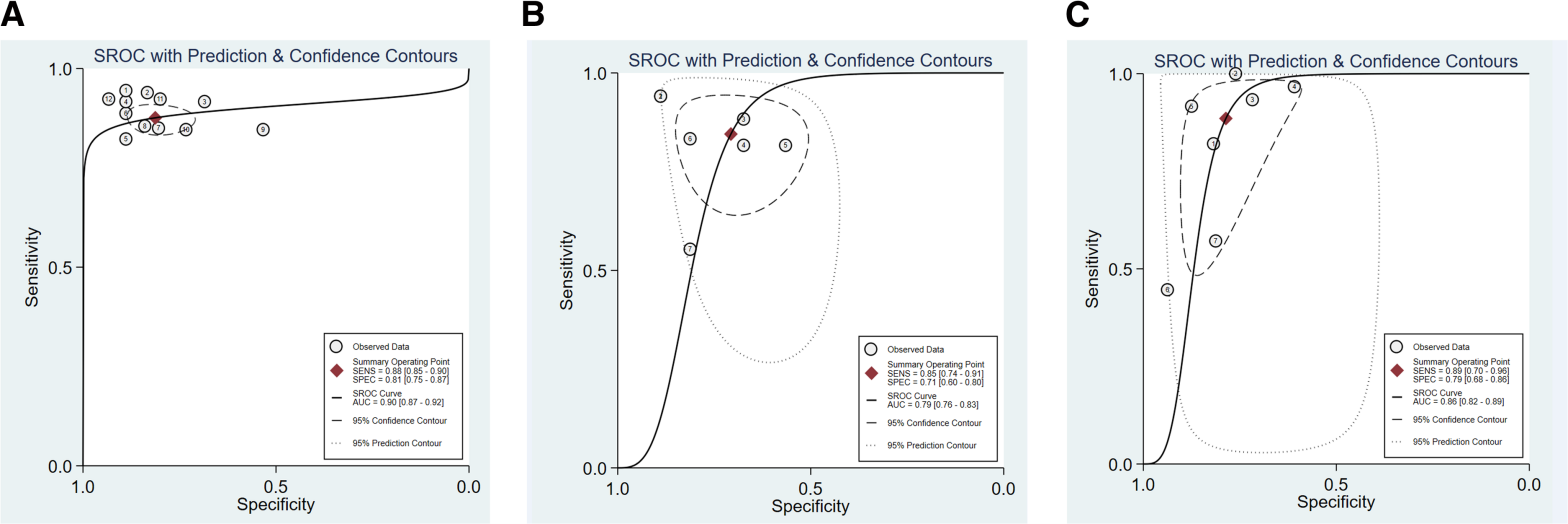
The area under the curve of summary receiver operating characteristic curves (AUSROC) of (**A**) LUS, (**B**) DE, and (**C**) DTF.

We assessed the pooled sensitivity, specificity, PLR, NLR, DOR, and AUC of the ROC for LUS, DE, and DTF to investigate the accuracy of ultrasound in predicting weaning outcomes (Table 2). The pooled sensitivity and specificity of LUS, DE, and DTF were 0.88 (95%CI, 0.85–0.90), 0.81 (95%CI, 0.75–0.87), 0.85 (95%CI, 0.74–0.91), 0.71 (95%CI, 0.60–0.80), 0.89 (95%CI, 0.70–0.96), and 0.79 (95%CI, 0.68–0.86), respectively (Figure 3). The pooled PLR and NLR of LUS, DE, and DTF were 4.68 (95%CI, 3.38–6.47), 0.15 (95%CI, 0.12–0.19), 2.88 (95%CI, 1.99–4.18), 0.22 (95%CI, 0.12–0.39), 4.14 (95%CI, 2.87–5.97) and 0.15 (95%CI, 0.05–0.40), respectively (Figure 4). The LUS, DE and DTF had the ability to accurately predict weaning, with DOR of 30.62 (95%CI, 18.86–49.72), 13.17 (95%CI, 5.65–30.71) and 28.35 (95%CI, 10.19–78.85), respectively. The area under the summary operator receiver characteristic curve (AUSROC) was shown in Figure 5, where LUS had the largest AUSROC of 0.90 (95%CI, 0.87–0.92), while DE and DTF were 0.79 (95%CI, 0.76–0.83) and 0.86 (95%CI, 0.82–0.89), respectively. In addition, heterogeneity was significant in the pooled sensitivity of DE and DTF (*I*^2 ^> 50%, *p* < 0.05).

**Table 2:** Diagnostic accuracy of LUS, DE and DTF by ultrasound.

	Sensitivity (95% CI)	Specificity (95% CI)	PLR (95% CI)	NLR (95% CI)	DOR (95% CI)	AUSROC
LUS	0.88 (0.85–0.90)	0.81 (0.75–0.87)	4.68 (3.38–6.47)	0.15 (0.12–0.19)	30.62 (18.86–49.72)	0.90 (0.87–0.92)
DE	0.85 (0.74–0.91)	0.71 (0.60–0.80)	2.88 (1.99–4.18)	0.22 (0.12–0.39)	13.17 (5.65–30.71)	0.79 (0.76–0.83)
DTF	0.89 (0.70–0.96)	0.79 (0.68–0.86)	4.14 (2.87–5.97)	0.15 (0.05–0.40)	28.35 (10.19–78.85)	0.86 (0.82–0.89)

LUS, lung ultrasound; DE, diaphragmatic excursion; DTF, diaphragm thickening fraction; PLR, Positive Likelihood Ratio; NLR, Negative Likelihood Ratio; 95% CI, 95% confidence interval; DOR, diagnostic odds ratio. AUSROC, area under the summary operator receiver characteristic curve.

### 3.4. Meta-regression and subgroup analyses

Due to a lack of data for DE and DTF, only sensitivity and specificity meta-regression analysis for LUS was performed, with the results presented in Supplementary Figure S2. Zone, weaning success rates (Pretest), threshold, and blind were all considered as factors influencing lung ultrasonography diagnostic accuracy. The sensitivity of LUS forecasts is affected by six Zone, weaning success rate more than 70%, LUS ≥ 15, and blind. Only six zones and weaning success rates greater than 70% have an effect on the specificity of LUS forecasts. In addition, we conducted subgroup analyses based on the meta-regression results, which were displayed in Table 3. Almost all subgroups demonstrated great high sensitivity, specificity, and DOR, indicating that LUS is a highly accurate technique for predicting infant weaning outcomes.

**Table 3:** The results of subgroup analysis of LUS.

Subgroups		Sensitivity (95% CI)	Specificity (95% CI)	PLR (95% CI)	NLR (95% CI)	DOR (95% CI)	AUSROC
Zone	Yes	0.88 (0.84–0.92)	0.81 (0.69–0.89)	4.60 (2.71–7.81)	0.15 (0.10–0.21)	31.28 (14.20–68.94)	0.90 (0.87–0.92)
	NO	0.87 (0.83–0.90)	0.83 (0.76–0.88)	4.99 (3.58–6.97)	0.15 (0.12–0.20)	32.35 (19.50–53.67)	0.92 (0.89–0.94)
Pretest	Yes	0.89 (0.85–0.92)	0.82 (0.72–0.90)	5.05 (3.02–8.46)	0.14 (0.10–0.19)	36.89 (17.15–79.33)	0.92 (0.89–0.94)
	NO	0.86 (0.81–0.90)	0.80 (0.72–0.86)	4.34 (3.01–6.24)	0.17 (0.12–0.25)	25.11 (13.97–45.14)	0.88 (0.85–0.91)
Threshold	Yes	0.89 (0.84–0.93)	0.84 (0.76–0.89)	5.55 (3.65–8.43)	0.13 (0.08–0.19)	43.54 (21.47–88.28)	0.90 (0.87–0.92)
	NO	0.86 (0.81–0.90)	0.79 (0.68–0.87)	4.13 (2.61–6.52)	0.18 (0.13–0.25)	22.96 (11.67–45.18)	0.87 (0.83–0.89)
Blind	Yes	0.90 (0.83–0.94)	0.83 (0.72–0.91)	5.38 (3.13–9.24)	0.12 (0.07–0.20)	43.72 (20.00–95.59)	0.93 (0.91–0.95)
	NO	0.87 (0.83–0.90)	0.80 (0.71–0.86)	4.30 (2.96–6.25)	0.16 (0.12–0.22)	26.16 (14.76–46.38)	0.90 (0.87–0.92)

PLR, Positive Likelihood Ratio; NLR, Negative Likelihood Ratio; 95% CI, 95% confidence interval; DOR, diagnostic odds ratio. AUSROC, area under the summary operator receiver characteristic curve.

## 4. Discussion

Although lung and diaphragmatic ultrasound have been frequently used to predict weaning in adult MV patients (32, 33, 34), their application in newborns has received little attention. In this meta-analysis, we discovered that lung and diaphragm ultrasound were also helpful techniques for predicting newborn weaning outcomes by examining the weaning outcomes of 828 infants who received MV. LUS performed the best overall, with sensitivity and specificity of 0.88 and 0.81, respectively. DTF outperformed DE in the diaphragm ultrasound evaluation index, and AUSROC was 0.86 and 0.79, respectively. It is important to note that the sensitivity of DE and DTF varies greatly. Moreover, some studies were highly susceptible to bias, particularly in patient selection, raising concerns about applicability.

LUS is an important method for evaluating lung ventilation loss, which represents a drop in lung capacity available for gas exchange and can be used to forecast EF (35, 36). Our data also shown a relationship between high LUS and EF, which is consistent with the findings for adults (37, 38). The unique truncation point, however, cannot be identified due to the variety of LUS methods, which include 6-zone, 10-zone, 12-zone, and 14-zone, but LUS has a high AUSROC of 0.90 (95%CI, 0.87–0.92) when assessed using the different cut-off points of each study. Although Jiang et al. (31) believe that prediction accuracy increases with partition size, the results of our subgroup analysis reveal that prediction accuracy for 6-zone and other zones is not significantly different, with AUSROC both above 0.90. We discovered that LUS was more accurate at predicting weaning in newborns than it was at predicting weaning in adults (AUSROC, 0.77) (15). Additionally, similarly to our work, LUS exhibited a good ability to predict weaning in several adult studies, with cut-off levels ranging from 10 to 14 (24, 39). In our subgroup analysis, subgroups with cut-off points higher than 15 had a DOR of 43.54 (95%CI, 21.47–88.28), greater than subgroups with cut-off points lower than 15, which had a DOR of 22.96 (95%CI, 11.67–45.18). The inclusion of groups with high extubation success rates and tight study methods were also sources of variability and variables with increased sensitivity, according to meta-regression.

DE and DTF are two key diaphragm ultrasonography measurements for predicting weaning results in MV patients, with DE being connected to inspiratory volume and DTF being a response measure of diaphragmatic inhalation effort (40, 41). DE and DTF worked well in adult studies, with greatest predictive values of 10–15 mm and 20%–36%, respectively (42). Except for one study, where the DTF cut-off point was chosen at 40.50% because the patients studied were generally healthy (28), the DTF cut-off values in the included studies were similar to those in adults. However, the ideal cut-off for DE in the studies we considered was often less than 10 mm, significantly smaller than the adult figure, with the exception of Gazi et al. (28), who used a cut-off of 12.15 mm. This could be because the diaphragm is a skeletal muscle whose thickness and strength fluctuate with age (43), and the children in Gazi et al. are much older than the children in the other included studies. In addition, infant diaphragms are not the same as adult diaphragms. The neonatal diaphragm, for example, is morphologically flat and implanted into the chest wall at a greater angle than the dome-shaped diaphragm in adults, resulting in a smaller apposition area and a limited range of displacement (44). In contrast to the adult diaphragm, which functions like a piston inside the ribs, the newborn diaphragm moves dorsally, like a bellows (45). Because the newborn diaphragm is structurally made of less anti-fatigue slow twitching (type I) fibers, reserve capacity is minimal and muscle fatigue is likely (46). When the amount of respiratory work rises, the accessory respiratory muscles (scalene and sternocleidomastoid) must be recruited to overcome tiredness (47).

Although the optimal cut-off points chosen by each study differed, our meta-analysis found that DE and DTF had good high sensitivity and moderate specificity for diagnosing infant weaning, with values of 0.85, 0.71, 0.89, and 0.79, respectively. In line with earlier studies, DTF outperformed DE in predicting MV weaning outcomes (48). Unfortunately, we were unable to perform meta-regression or subgroup analysis of DE and DTF results to determine the source of their heterogeneity due to the short number of papers available. We still consider Yao's studies as a significant source of heterogeneity because their sensitivity was 0.45 to 0.57, which was much lower than in other researches. Therefore, we feel that the capabilities of diaphragm ultrasound are underappreciated and that it could play a more prominent role in predicting infant MV weaning outcomes.

Ana M. Llamas-Llvarez et al. (15) found in a systematic analysis of adults that DTF performed better than DE in predicting patient weaning outcomes, and our investigation supported this finding for infants. Even if their findings also explain the power of LUS, they feel that this information should be viewed with caution because they were unable to undertake a thorough investigation of the capabilities of LUS due to limitations in the included literature. We can categorically state that lung ultrasonography is effective at predicting weaning outcomes because we included enough material to enable us to carry out in-depth subgroup analysis. Overall, our study found that both diaphragmatic and lung ultrasound had good accuracy and can be used to forecast the results of newborn weaning.

The lungs and diaphragm are coordinated during breathing motions because the diaphragm acts as a brake at the end of expiration to maintain end-expiratory lung volume (EELV) and prevent lung collapse (49). Patients who receive positive end-expiratory pressure ventilation have their diaphragmatic myofibers remodeled over time with the help of MV, whereas when weaned, sudden changes in EELV when the diaphragm does not have time to respond reduce inspiratory muscle strength and lead to weaning failure (50). Prolonged MV reduces diaphragm thickness, and a study in adults found that a thinner diaphragm is associated with weaning failure (51). A recent agreement on diaphragm ultrasonography in critically ill patients recommends a thickness drop of more than 10% as the cut-off for atrophy (52). Furthermore, excessive diaphragmatic effort can still result in self-inflicted lung injury since spontaneous breathing efforts can cause the lungs to stretch abruptly during inflation, even if tidal volume is not increased (53). Therefore, fresh ideas for lung and diaphragm protective breathing have been put forth (54). Although preliminary clinical trials are still being conducted, we may anticipate further study on lung and diaphragm interactions to increase the likelihood that weaning will be successful in the future.

Our meta-analysis did have certain limitations. First, the infant ages in the research we included varied greatly, thus there could be a consistent effect on the accuracy of the results. Second, differences in diaphragmatic ultrasonography procedures, which might be difficult to identify and harmonize, may have an adverse effect on the outcomes. Moreover, we only made a preliminary conclusion that pulmonary and diaphragm ultrasound are useful instruments for measuring newborn weaning with MV, but there is no consensus on acceptable cut-off values. Finally, because of the small number of studies included, we were unable to investigate the sources of heterogeneity in diaphragm ultrasound measurements.

## 5. Conclusion

The findings of this meta-analysis demonstrate that high LUS, low DE, and low DTF all indicate EF, implying that lung and diaphragm ultrasound, particularly LUS performance, is an excellent technique for predicting weaning outcomes in infants with MV. Subgroup analysis of diaphragm ultrasonography is also required to establish its predictive accuracy under different definitions and measurement settings.

## Data Availability

The original contributions presented in the study are included in the article/**Supplementary Material**, further inquiries can be directed to the corresponding author.
