# Supplementary material for: Accuracy of lung and diaphragm ultrasound in predicting infant weaning outcomes: a systematic review and meta-analysis
Source: Front Pediatr. 2023 Sep 6;11:1211306. doi: 10.3389/fped.2023.1211306 (PMC10511769; doi:10.3389/fped.2023.1211306)
Supplement: Supplementary file 1 [file Table1.docx]

Supplementary Material

Accuracy of lung and diaphragm ultrasound in predicting infant weaning outcomes: a systematic review and meta-analysis

**Yang Gao^1^, Hong Yin^1^, Meihuan Wang^2^, Yuehua Gao^2*^**

**^1^ ^Department of Ultrasound, Shandong Provincial Maternal and Child Health Care Hospital, Jinan, Shandong, China.^**

**^2 Department of Ultrasound, Shandong Provincial Hospital affiliated to Shandong First Medical University, Jinan, Shandong, China.^**

*** Correspondence:** **Yuehua Gao (longshe7@126.com)**


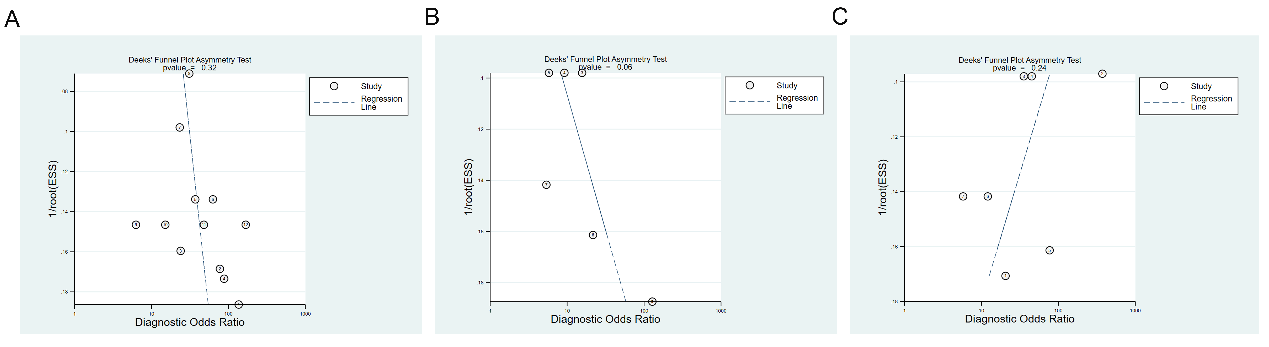


Figure S1. Deeks’ funnel plot asymmetry test of (A) LUS, (B) DE, and (C) DTF.


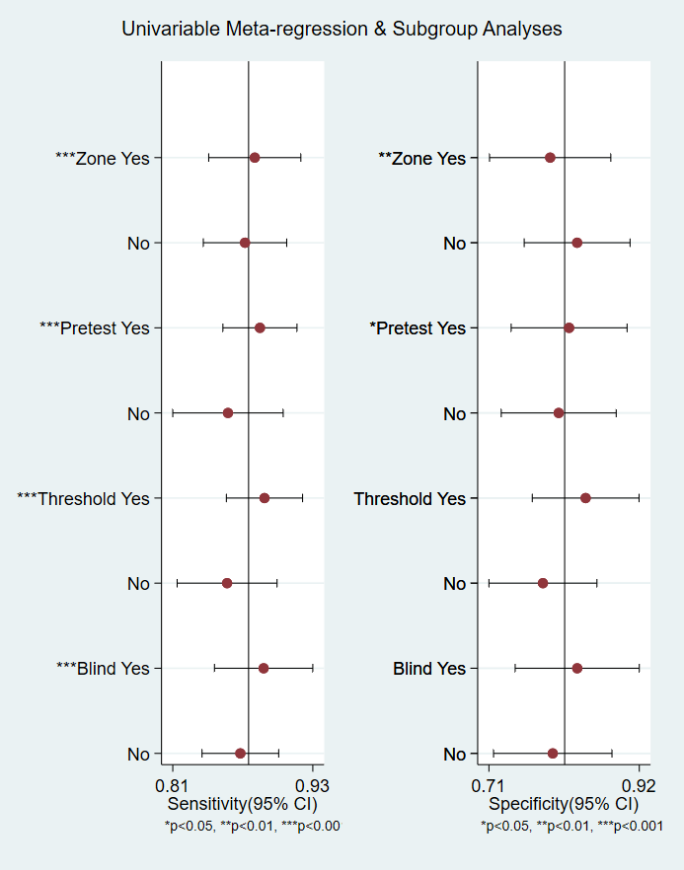


Figure S2. Meta-regression to explore heterogeneity between studies for LUS

Zone: Yes = 6-Zone, No = other; Pretest: Yes = common prevalence of extubation success, No = low prevalence of extubation success (< 70%); Threshold: Yes = LUS scores ≥ 15; No = other; Blind: Yes = The study process was explicitly blinded, No = other.
